# Comparison between Magneto-Dynamic, Piezoelectric, and Conventional Surgery for Dental Extractions: A Pilot Study

**DOI:** 10.3390/dj11030060

**Published:** 2023-02-23

**Authors:** Francesco Bennardo, Selene Barone, Camillo Vocaturo, Dorin Nicolae Gheorghe, Giorgio Cosentini, Alessandro Antonelli, Amerigo Giudice

**Affiliations:** 1 School of Dentistry, Department of Health Sciences, Magna Graecia University of Catanzaro, Viale Europa, 88100 Catanzaro, Italy; 2 Independent Researcher, Via Nicola Serra 80, 87100 Cosenza, Italy; 3 Department of Periodontology, Faculty of Dental Medicine, University of Medicine and Pharmacy of Craiova, 200349 Craiova, Romania; 4 Life Sciences PhD School, Magna Graecia University of Catanzaro, Viale Europa, 88100 Catanzaro, Italy

**Keywords:** Magnetic Mallet, piezosurgery, tooth extraction

## Abstract

This pilot split-mouth study aimed to evaluate and compare early postoperative discomfort and wound healing outcomes in post-extraction sockets after dental extraction performed with a Magnetic Mallet (MM), piezosurgery, and conventional instruments (EudraCT 2022-003135-25). Twenty-two patients requiring the extraction of three non-adjacent teeth were included. Each tooth was randomly assigned to a specific treatment (control, MM, or piezosurgery). Outcome measures were the severity of symptoms after surgery, wound healing assessed at the 10-days follow-up visit, and the time taken to complete each procedure (excluding suturing). Two-way ANOVA and Tukey’s multiple comparisons tests were performed to evaluate eventual differences between groups. There were no statistically significant differences between the compared methods in postoperative pain and healing, and no additional complications were reported. MM required significantly less time to perform a tooth extraction, followed by conventional instruments and piezosurgery, in increasing order (*p* < 0.05). Overall, the present findings suggest the use of MM and piezosurgery as valid options for dental extractions. Further randomized controlled studies are needed to confirm and extend this study’s results, facilitating the selection of the optimal method for an individual patient depending on the patient’s needs and preferences.

## 1. Introduction

A traumatic dental extraction may lead to complications related to hard and soft tissue damage [1]. An appropriate surgical technique is necessary to obtain tissue healing for the functional and aesthetic stability of prosthetic restorations [2]. The use of several tools for tooth extraction has been described. In recent decades, laser, piezoelectric, and magneto-dynamic technologies have been used in oral surgery; however, there is a paucity of clinical evidence regarding their use for dental extraction. The evaluation of these technologies is complex, since protocols and methods are changing as innovations are used in clinical practice [1,3]. To avoid broad acceptance with insufficient evidence and to encourage innovation backed by enough data, new surgical procedures must be evaluated as soon as possible [4]. Currently, the standard dental extraction technique involves elevators, periotomes, and forceps, which operate on the principle of socket expansion. These instruments are often associated with tissue trauma and increased postoperative morbidity [5]. The surgical extraction of ankylosed or devitalized teeth is one of the most challenging oral surgical procedures. In the case of periodontal ligament (PDL) atrophy or unfavorable anatomical conditions, in addition to odontotomy, it is necessary to create spaces between roots and the alveolar bone to complete the extraction. Conventional surgery involves the use of burs inserted into a rotary handpiece with or without the elevation of a flap [6]. Recently, piezoelectric technology has proved to be a valid alternative to conventional surgery. Piezoelectric surgery uses ultrasounds to perform bone tissue cuts to preserve nerves, vessels, and soft tissues. The precision of cutting, the cavitation effect that reduces bleeding, and the production of lesser bone necrosis are the main technical features of ultrasonic surgery [7,8]. Several authors have discussed the application of magneto-dynamic technology in dental extractions. Taking advantage of electromagnetism’s basic principles, magneto-dynamic technology applies controlled forces to a subject during a very short time of impact. Procedures are safe for patients and surgeons because of the control and constancy of the applied pressures. The first electrified mallet for gold fillings of dental cavities was invented by Bonwill in 1873. The Magnetic Mallet (MM) device uses magneto-dynamic technology. It consists of a handpiece powered by a central managing system. The handpiece, which pushes a shock wave on its tip with different powers and delivers forces by application timing (80 μs impact) and in accordance with various surgical techniques, could use several attached inserts (periotomes, osteotomes, ridge expanders). However, it is not obvious whether these applications have any scientific support [5]. This pilot split-mouth study aimed to evaluate and compare early postoperative discomfort and wound healing outcomes in post-extraction sockets after dental extraction performed with different instruments. MM, piezosurgery, and conventional instruments were compared. The null hypothesis was no difference between the three surgical techniques in terms of postoperative pain, healing, the time taken to complete each procedure, and additional complications.

## 2. Materials and Methods

The present article is reported according to the Consolidated Standards of Reporting Trials (CONSORT) statement and its extension for within-person randomized studies [9].

### 2.1. Study Design

According to the Declaration of Helsinki on medical protocol and ethics, the Regional Ethical Review Board of Central Calabria (reference for the Magna Graecia University of Catanzaro, Catanzaro, Italy) approved the study (n. 463/2020). It was designed as a within-person randomized split-mouth pilot study (EudraCT 2022-003135-25).

### 2.2. Study Sample

Participants were recruited in the Academic Hospital of Magna Graecia University of Catanzaro, Italy. Patients who needed the extraction of three non-adjacent teeth with similar characteristics (single-rooted, incisors, and canines; multi-rooted, premolars, and molars), following the “SIdCO” (Italian Society of Oral Surgery) position statement on teeth extractions (teeth that cannot be restored or recovered with periodontal or orthodontic–prosthetic treatments) [10], were included. The exclusion criteria were as follows: persons under the age of 18; acute infection in any of the selected teeth; severe periodontal disease; smokers (>5 cigarettes per day); a history of antiresorptive therapy or maxillofacial irradiations; patients with diabetes mellitus, chronic liver disease, immune system dysfunction, or hematological disease; pregnancy or breastfeeding. Informed consent was obtained from all enrolled patients after being adequately informed of the treatment’s risks and potential benefits. Considering the study type, the number of treated patients was used to calculate the power of this pilot study given an effect size of 0.5, and a type I error of 0.05, using G* Power (G* Power version 3.1.9.7, G* Power Team, Heinrich Heine Universität Düsseldorf, Germany).

### 2.3. Procedure

Before extraction, the patients received a single dose of prophylactic antibiotic 1 h before the intervention: 2 g of amoxicillin or 600 mg of clindamycin if allergic to penicillin. The patients rinsed with 0.2% chlorhexidine mouthwash for 1 min before the intervention. Local anesthesia was delivered as required, using mepivacaine hydrochloride 20 mg/mL 1:100,000 adrenalin (Optocain, Molteni Dental, Milano, Italy). All procedures were performed by the same operator in a single session. Three interventions were compared, as described in Table 1. Each tooth was assigned to a specific treatment (control, MM, or piezosurgery) by choosing between identical, opaque envelopes containing different combinations. Teeth extractions were performed as atraumatically as possible. Flapless extractions were attempted; however, if necessary, flaps were elevated. Granulation tissues were removed from extraction sockets with an alveolar spoon. After extractions, all sockets were sutured with a 4-0 polyglactin suture (Vycril, Ethicon, J&J, Somerville, NJ, USA). The time taken to complete each individual procedure was measured by an operator assistant (excluding suturing). In case of pain, the patients were instructed to take paracetamol at 1 g or metamizole at 500 mg if allergic to paracetamol. The patients were instructed not to eat or drink for at least 90 min after surgery, brush, or use any mouthwash for the first postoperative day. Chlorhexidine mouthwash at 0.2% had to be used for 1 min twice a day starting the second postoperative day until control.

### 2.4. Outcome Assessment

Ten days after teeth extraction, all patients were interviewed and checked for soft tissue healing by a blinded clinician. Sutures were also removed on the same day.

The primary outcome measures were:

- Pain assessment

Postoperative pain was assessed through a visual analog scale (VAS). The patients were asked to grade the severity of their symptoms in numbers from 0 (no discomfort/pain) to 10 (very severe discomfort/pain) for each site of extraction;

- Healing assessment

Healing was assessed through the wound healing index (WHI). WHI was assessed at the 10-days follow-up visit by a blinded operator using the following scoring system, as reported by a previous study [11]:

1. Complete wound closure without the presence of fibrin;
2. Complete wound closure with the presence of fibrin;
3. Incomplete wound closure (dehiscence);
4. Incomplete wound closure (necrosis).

The secondary outcome measures were:

- Time to complete each individual procedure (excluding suturing; measured in second);
- Any additional complication (oroantral communication, root fracture, bleeding, dry socket or alveolitis, abscess, fistula, nerve injury).

### 2.5. Data Collection, Processing, and Statistical Analysis

The collected data were managed and processed by a third clinician who was unaware of which intervention was assigned to each site. The study groups were analyzed using descriptive statistics (absolute and relative frequencies). Mean, median, mode, and standard deviation were used to describe the variables. Two-way ANOVA (or mixed model in the case of missing values) and Tukey’s multiple comparisons tests were performed to evaluate eventual differences between groups. For single- and multi-rooted teeth, we performed subgroup analyses.

The level of significance was set at *p* < 0.05. The statistical analysis was performed by using the STATA software program (STATA Release 14; STATA Corporation, College Station, TX, USA) and GraphPad Prism 9 (GraphPad Prism version 9.2.0, GraphPad Software, San Diego, CA, USA). The null hypothesis was no difference between the three treatments performed.

## 3. Results

### 3.1. Study Sample

**Figure 1:**
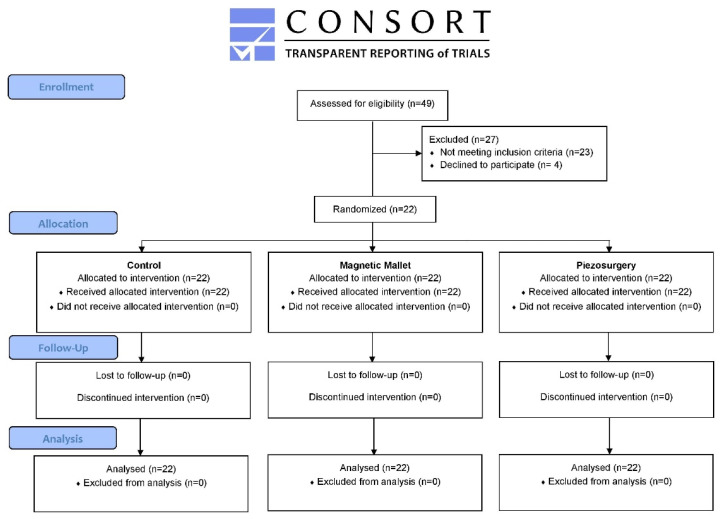
CONSORT flow diagram.

From January 2022 to July 2022, forty-nine patients who needed the extraction of three non-adjacent teeth were screened for eligibility: twenty-three patients did not meet the inclusion criteria and four declined to participate. A total of twenty-two patients were included in this study and had three non-adjacent teeth randomly allocated to the three tested interventions. The effective power of the study was 73% (calculated considering the patients actually treated and the parameters reported in the Section 2). Five patients needed single-rooted teeth extraction, and 17 needed multi-rooted teeth extraction. The data presented are summarized in the CONSORT flow diagram (Figure 1). The mean age of patients was 43.77 ± 12.69 (range 25–64 years); eight were female, and fourteen were males, with a male-to-female ratio of 1.75:1.

### 3.2. Outcomes

**Figure 2:**
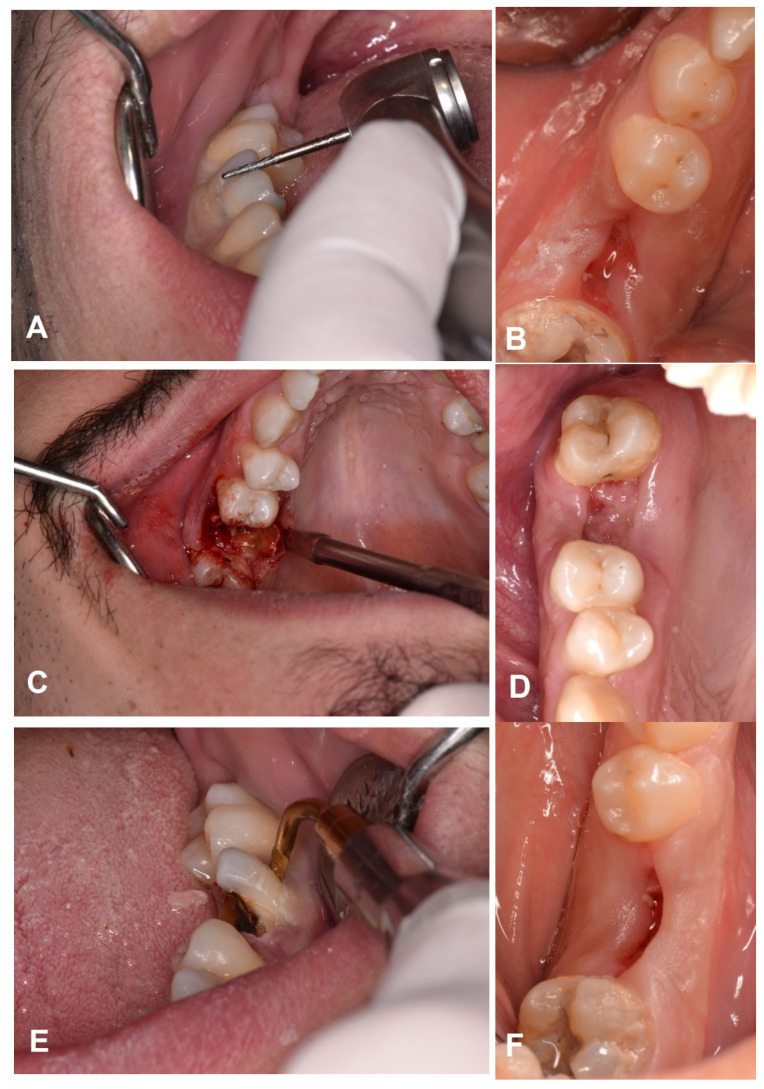
(**A**) Extraction of right mandibular first molar: control intervention (turbine with surgical bur). (**B**) Healing after suture removal. (**C**) Extraction of right maxillary first molar: Magnetic Mallet (EXTR 1) (**D**) Healing after ten days. (**E**) Extraction of right mandibular first molar: Piezosurgery (EX 1) (**F**) Healing at the follow-up visit.

All procedures were completed for twenty-two patients. All sites were treated according to the allocated interventions. Figure 2 reports one case as an example.

No flap was elevated, no patient dropped out, and the data of all patients were evaluated in the statistical analyses. No complications were reported. No deviations from the operative protocol occurred. Teeth positions randomly allocated to each group are presented in Table 2. 

### 3.3. Data Analysis

The two-way ANOVA test rejected the null hypothesis of no difference between treatment groups and subgroups (*p* < 0.05). The results of Tukey’s multiple comparisons test are reported in the following sub-sections according to the outcome analyzed (pain, healing, time).

#### 3.3.1. Pain Assessment

**Figure 3:**
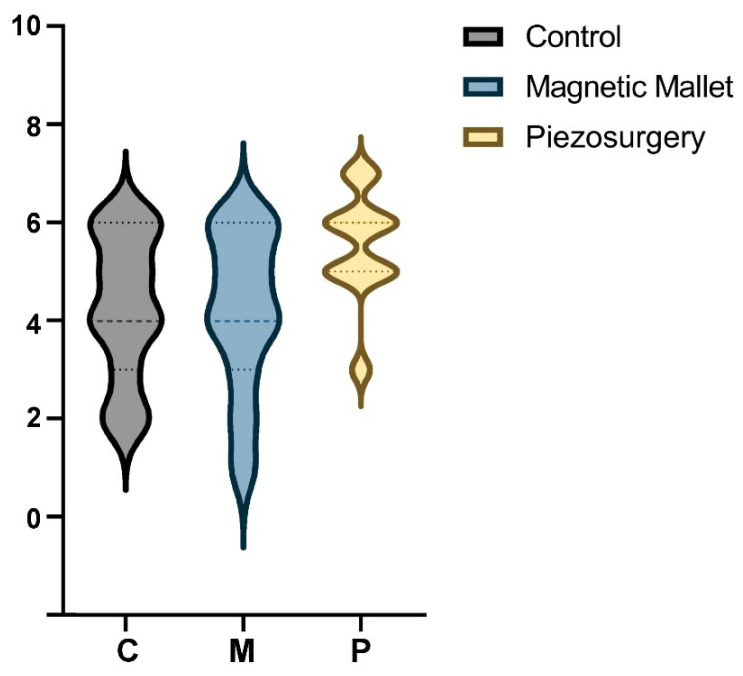
Violin plot of pain distribution (VAS).

The pain assessment scores’ (Figure 3) mean was 4.27 ± 1.45 (range 3–6) in the control group, 4.18 ± 1.62 (range 3–6) in the MM group, and 5.54 ± 1.10 (range 5–6) in the piezosurgery group. Tukey’s multiple comparisons showed no difference between groups and subgroups (*p* > 0.05). 

#### 3.3.2. Healing Assessment

**Figure 4:**
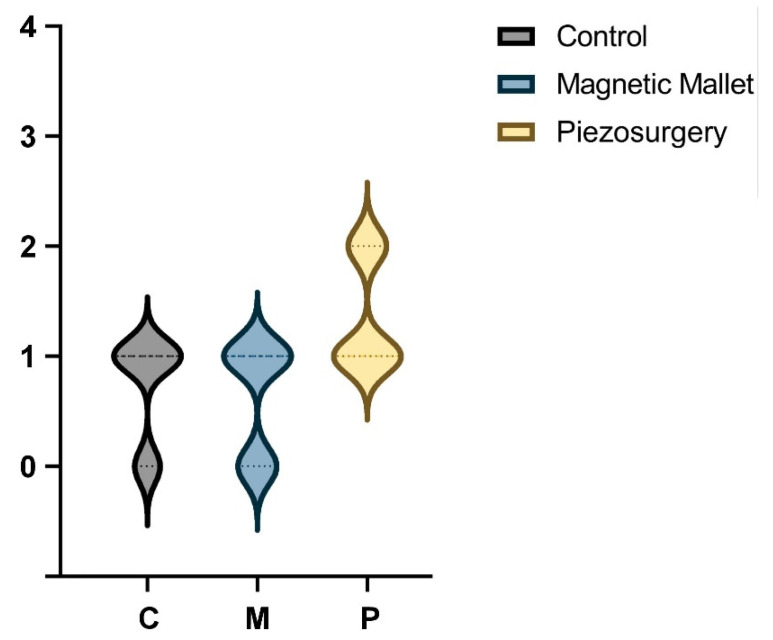
Violin plot of healing distribution (WHI).

The healing assessment scores’ (Figure 4) median and mode were 1 in all groups (control group range 0–1; MM group range 0–1; piezosurgery group range 5–6). Tukey’s multiple comparisons showed no difference between groups and subgroups (*p* > 0.05).

#### 3.3.3. Time to Complete Each Individual Procedure

**Figure 5:**
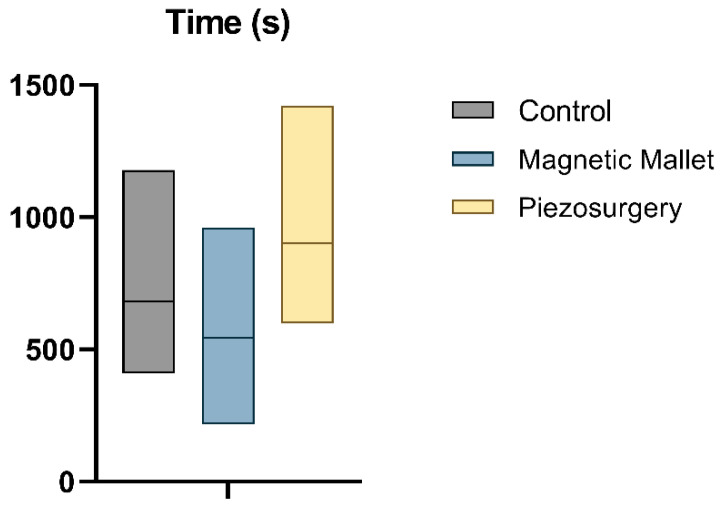
Box plot of time.

The average time necessary to complete each procedure (Figure 5) was 682.3 ± 289.3 s in the control group, 544.9 ± 268.2 in the MM group, and 901.0 ± 293.4 (range 5–6) in the piezosurgery group. Tukey’s multiple comparisons showed a difference between all groups: the time necessary to complete the procedure with MM was significantly lower compared to both the control (*p* = 0.02) and piezosurgery (*p* < 0.0001) groups. The use of piezosurgery required a significantly longer time compared to the control (*p* < 0.0001). Tukey’s multiple comparisons showed different results considering single- and multi-rooted subgroups: the results were confirmed for multi-rooted teeth (*p* < 0.05) but not for single-rooted teeth (*p* > 0.05).

#### 3.3.4. Any Additional Complication

No oroantral communication, no root fracture, no bleeding, no dry socket or alveolitis, no abscesses, no fistula, and no nerve injury were reported.

## 4. Discussion

The aim of this pilot study was to evaluate postoperative discomfort and wound healing outcomes after dental extraction performed with MM and piezosurgery compared to traditional tools. To the best of the authors’ knowledge, this is the first study aiming to compare the efficacy of these devices in reducing discomfort and improving tissue healing. According to our data, it is possible to affirm that both devices were reliable in all interventions. The statistical analysis suggested that there were no significant differences between the compared interventions, except for the time required to complete surgeries unfavorable for piezosurgery. 

Depending on the method employed to remove the tooth, the PDL may sustain trauma ranging from the simple surgical extraction of a single-rooted tooth using periotomes, elevators, and forceps, to the complex surgical extraction involving the reflection of a mucoperiosteal flap and bone removal. Traditional methods used to increase space around the teeth include tapping a periotome or using rotary instruments. In the first case, there is a risk of alveolar bone fracture, benign paroxysmal positional vertigo (BPPV), and temporomandibular disorders due to poor control of the applied forces. Otherwise, deboning using a traditional rotary handpiece may involve the elevation of a mucoperiosteal flap to prevent soft tissue injury. Both methods may affect postoperative discomfort and tissue healing [6]. 

Since the principle of tooth extraction is socket expansion, it is impossible to avoid bone trauma, and even a successful extraction utilizing elevators or periotomes will cause some soft tissue trauma. Several of the least-invasive tooth extraction methods using modern technology (vertical extraction, piezoelectric, magneto-dynamic) have been described, yet no extraction method can be fully atraumatic [3,12]. While the efficacy of piezosurgery in dental extractions was already confirmed by several studies regarding impacted third molars [13,14], data are limited for MM [5].

Piezosurgery was introduced almost 20 years ago in dentistry [15]. Its features allowed the selective cutting of mineralized tissue and the preservation of the integrity of mucosa, vessels, and nerves in case of accidental contact. Piezoelectric crystal creates a micrometric vibration of the device’s active tip via ultrasonic wave modulation, enabling incredibly accurate cutting and improved intraoperative control [16]. Microstreaming and the cavitation effect, two additional PBS features, also enhance the surgical field conditions during ultrasonic osteotomy. This continuous fluid-swirling movement is caused by active tip vibration and favors the mechanical action of debris removal. During osteotomy, the cavitation effect, a physical phenomenon produced by the collapse of gas bubbles inside terminal blood arteries, generates a hemostatic effect that improves intraoperative vision [8]. These characteristics allowed a rapid spread of this technology in all surgical branches, from orthopedics to neurosurgery, including head and neck surgery [17,18,19].

Piezosurgery has been widely applied in oral and maxillofacial surgery such as for impacted teeth extraction, and regenerative surgery, such as sinus augmentation, ridge expansion, and bone harvesting [7,15,20]. However, the data on the use of piezosurgery in non-impacted teeth extraction are limited [6].

In accordance with recent meta-analyses, the duration of surgery for impacted third-molar extraction was found to be significantly shorter in the control group (rotary instruments) compared to piezosurgery [20,21]. These results agree with the findings reported in this pilot study about the time required for interventions. Regarding other parameters analyzed in the present study, despite the longer duration of surgery, postoperative morbidity parameters were significantly lower in the piezosurgery group, compared to the control group, only until the seventh postoperative day. No data were available regarding wound healing.

Anyway, flapless surgery has the advantages of reducing post-surgical morbidity effects and accelerating healing [22]. For this reason, the clinical scenario analysis in this study could limit the potential benefit of piezosurgery described for the removal of impacted third molars.

Cai et al. aimed to investigate the use of piezosurgery with a flapless approach to increase bone space during tooth extraction and assess its effectiveness in terms of postoperative complications. A total of 140 patients underwent flapless teeth extraction through piezosurgery. No complications were reported by the authors, except for two cases with buccal mucosal injury probably caused by heat piezosurgery tip. The prevention of root and alveolar bone fractures during surgery, and soft tissue preservation, were all benefits of teeth extraction using piezosurgery with a flapless approach [6]. These results agree with the findings reported in this pilot study. 

MM is a patented device, which uses electromagnetic impact to generate a high-intensity, transient impact force that causes plastic deformation of the bone. When compared to the traditional chisel and mallet or bur used for bone cutting, MM has many advantages, since it prevents the applied forces from having an impact on the entire craniofacial complex, preventing BPPV, and with less patient discomfort [23]. A controlled fracture and displacement of the cortical bone as well as increased bone tissue density along the walls are the results of the longitudinal movement that the MM handpiece imparts along the osteotome’s axis. This action acts upon and forces the internal wall of the hole outward radially [24,25,26].

A few studies on the use of MM for dental extractions were observed in the literature research, but only one retrospective clinical study was exclusively devoted to this subject. Crespi et al. reported 427 teeth extractions using MM in 156 patients. According to the authors, the axial movements applied to the blade’s tip allowed the root to be separated from the surrounding alveolar bone while minimizing damage to nearby bone and gingival tissues. The authors observed no root or cortical bone fractures or impaired soft tissue healing [27]. These results agree with the findings reported in this pilot study. No other results are available in the literature about clinical studies focused on the efficacy of MM for dental extractions, except studies related to the timing of implant insertion and socket healing with the preservation of reactive soft tissues in which teeth extractions were performed with MM without any complication [28].

Given the benefits mentioned, the use of MM could optimize the clinical workflow for the isolation of stem cells from periodontal ligament and other dental sources [29,30].

The use of specific surgical burs, saws, laser, or piezoelectric devices was described to increase the amount of bone before or simultaneously with implant insertion [5].

Histological tests conducted by Schierano et al. revealed that magneto-dynamic technology can considerably increase the amount of newly generated bone tissue and osteoblasts in comparison to drills. The intrinsic ability of MM to osteocondensate bone tissue can positively affect bone healing and the primary stability of dental implants. In addition, an increase in cytokines related to osteogenesis has been observed in these sites, suggesting a positive trend in bone maturation and secondary implant stability [31]. Feher et al. set up a study on implant site preparation with MM both in edentulous ridges and in fresh sockets. They evaluated the implant stability quotient (ISQ) values of implants inserted in condensed bone sites prepared with MM. Before the use of MM, a traditional drill was used to prepare the pilot hole of 2.2 mm in diameter and 8 mm in depth. Their main finding related to the use of magneto-dynamic technology was that implant site preparation with MM led to higher ISQ but not higher insertion torque values. For these reasons, the use of MM for implant site preparation needs further investigations [32]. MM could be used not only for implant site preparation, but also for crestal sinus lift and ridge expansion [5].

In the past two years, recommendations have been made to reduce the risk of viral transmission following the emergence of the severe acute respiratory syndrome coronavirus 2 (SARS-CoV-2) pandemic, which includes reducing the formation of aerosols during dental treatments [33,34,35]. 

Chien et al. recently reported a technical note about lower third molar extraction us-ing a mallet and chisel to reduce aerosolizing during surgical interventions. To separate the tooth from the surrounding bone, the mallet has to be tapped on the head of the chisel. This procedure may result in BPPV and may put the patient through needless suffering. Additionally, the surgeon might experience discomfort using this procedure [36]. Considering the issue with aerosol-generating procedures and exposure to SARS-CoV-2, using the MM for surgical tooth extraction as a strategy to reduce aerosol exposure is a possibility, instead of using conventional instruments and piezosurgery [5].

The limitations of this study include the study design, the low sample size, no evalu-ation of teeth extraction according to jaw type, no three-dimensional radiological evalua-tion of bone healing, VAS for patient discomfort evaluation, and subjective assessment methods for wound healing evaluation.

Among these, a fundamental criticism that should be made with respect to the VAS approach is that it provides information on “how much”, but it does not tell us exactly “how much of what”, as reported by Franchignoni et al. [37]. However, the study design and the simultaneous application of the three surgical techniques should have reduced any bias related to using VAS in evaluating postoperative discomfort, even if some patients took painkillers.

Regarding the influence of jaw type on healing, maxillary bone mineral density is lower than the mandible and, at the same time, posterior maxillary bone mineral density is lower than the anterior maxilla [38]. The authors struggled to recruit patients requiring the extraction of three non-adjacent teeth with similar characteristics (single-rooted vs. multi-rooted) in the same jaw (maxillary or mandible). Furthermore, in the case of non-adjacent teeth extractions in the same sextant, the patients may have had difficulty pinpointing the exact socket causing the discomfort or pain. A within-person study is probably not feasible for comparing three different techniques. Still, a split-mouth study comparing two approaches for the extraction of similar teeth on the same jaw must be considered in future research protocols.

## 5. Conclusions

There were no statistically significant differences between the three surgical techniques analyzed (MM, piezosurgery, traditional) for teeth extractions in terms of postoperative pain and healing, and no additional complications were reported. However, MM required significantly less time to perform a tooth extraction, followed by conventional instruments and piezosurgery, in increasing order (*p* < 0.05). Overall, the present findings suggest the use of MM and piezosurgery as valid options for dental extractions. Further randomized controlled studies are needed to confirm and extend this study’s results, facilitating the selection of the optimal method for an individual patient depending on the patient’s needs and preferences.

## Figures and Tables

**Table 1:** Interventions compared in this study.

Arm	Treatment
Control (C)	Periotomes to remove PDL fibers Contra-angle handpiece/turbine with surgical burs to remove periradicular bone Elevators and extraction forceps for luxation
Magnetic Mallet (M)	Magnetic Mallet^®^ handpiece with dedicated tips (EXTR 1, EXTR 2, EXTR 3, EXTR 4, EXTR 5; Osseotouch, Gallarate, VA, Italy) for removing PDL fibers, periradicular bone, and luxation
Piezosurgery (P)	Piezosurgery^®^ handpiece with dedicated ultrasonic tips (EX 1, EX 2, EX 3; Mectron, Carasco, GE, Italy) to remove periodontal ligament fibers and periradicular bone Elevators and extraction forceps for luxation

N.B. For multi-rooted teeth, odontotomy was performed with rotary instruments in all groups. After luxation, if necessary, extraction forceps were used to complete dental extraction. Same post-operative recommendations.

**Table 2:** Distribution of extracted teeth.

Subgroups	Extracted Teeth	Control (n = 22)	Magnetic Mallet (n = 22)	Piezosurgery (n = 22)
Single-rooted (5 patients)	Incisors	3 Mandible 1, Maxilla 2	3 Mandible 2, Maxilla 1	4 Mandible 2, Maxilla 2
	Canines	2 Mandible 1, Maxilla 1	2 Mandible 1, Maxilla 1	1 Mandible -, Maxilla 1
Multi-rooted (17 patients)	Premolars	9 Mandible 4, Maxilla 5	8 Mandible 3, Maxilla 5	7 Mandible 4, Maxilla 3
	Molars	8 Mandible 4, Maxilla 4	9 Mandible 3, Maxilla 5	10 Mandible 6, Maxilla 4

## Data Availability

The data presented in this study are available on request from the corresponding author.

## References

[1] Hong B., Bulsara Y., Gorecki P., Dietrich T. (2018). Minimally invasive vertical versus conventional tooth extraction: An interrupted time series study. J. Am. Dent. Assoc..

[2] Atieh M.A., Alsabeeha N.H.M., Tawse-Smith A., Duncan W.J. (2018). Piezoelectric versus conventional implant site preparation: A systematic review and meta-analysis. Clin. Implant. Dent. Relat. Res..

[3] Saund D., Dietrich T. (2013). Minimally-invasive tooth extraction: Doorknobs and strings revisited!. Dent. Update.

[4] Ergina P.L., Cook J., Blazeby J., Boutron I., Clavien P.-A., Reeves B.C., Seiler C.M. (2009). Challenges in evaluating surgical innovation. Lancet.

[5] Bennardo F., Barone S., Vocaturo C., Nucci L., Antonelli A., Giudice A. (2022). Usefulness of Magnetic Mallet in Oral Surgery and Implantology: A Systematic Review. J. Pers. Med..

[6] Cai Y., Sun R., Zhao J.H. (2018). Flapless boning to increase space by piezosurgery: A novel mini-invasive strategy for teeth extraction. A retrospective study. Medicine.

[7] Vercellotti T., De Paoli S., Nevins M. (2001). The piezoelectric bony window osteotomy and sinus membrane elevation: Introduction of a new technique for simplification of the sinus augmentation procedure. Int. J. Periodontics Restor. Dent..

[8] Vercellotti T. (2004). Technological characteristics and clinical indications of piezoelectric bone surgery. Minerva Stomatol..

[9] Pandis N., Chung B., Scherer R.W., Elbourne D., Altman D.G. (2017). CONSORT 2010 statement: Extension checklist for reporting within person randomised trials. BMJ.

[10] SIDCO Position statement su “appropriatezza in chirurgia estrattiva”: Criteri decisionali endodontici, restaurativi, parodontali. https://www.sidcoinforma.it/download/position.pdf.

[11] Giudice A., Esposito M., Bennardo F., Brancaccio Y., Buti J., Fortunato L. (2019). Dental extractions for patients on oral antiplatelet: A within-person randomised controlled trial comparing haemostatic plugs, advanced-platelet-rich fibrin (A-PRF+) plugs, leukocyte- and platelet-rich fibrin (L-PRF) plugs and suturing alone. Int. J. Oral Implantol..

[12] Barone S., Antonelli A., Averta F., Diodati F., Muraca D., Bennardo F., Giudice A. (2021). Does Mandibular Gonial Angle Influence the Eruption Pattern of the Lower Third Molar? A Three-Dimensional Study. J. Clin. Med..

[13] Sortino F., Pedullà E., Masoli V. (2008). The piezoelectric and rotatory osteotomy technique in impacted third molar surgery: Comparison of postoperative recovery. J. Oral Maxillofac. Surg..

[14] Tsai S.J., Chen Y.L., Chang H.H., Shyu Y.C., Lin C.P. (2012). Effect of piezoelectric instruments on healing propensity of alveolar sockets following mandibular third molar extraction. J. Dent. Sci..

[15] Vercellotti T. (2000). Piezoelectric surgery in implantology: A case report—A new piezoelectric ridge expansion technique. Int. J. Periodontics Restor. Dent..

[16] Stacchi C., Berton F., Turco G., Franco M., Navarra C.O., Andolsek F., Maglione M., Di Lenarda R. (2016). Micromorphometric analysis of bone blocks harvested with eight different ultrasonic and sonic devices for osseous surgery. J. Craniomaxillofac. Surg..

[17] Zhang Y., Wang C., Zhou S., Jiang W., Liu Z., Xu L. (2017). A comparison review on orthopedic surgery using piezosurgery and conventional tools. Procedia Cirp..

[18] Massimi L., Rapisarda A., Bianchi F., Frassanito P., Tamburrini G., Pelo S., Caldarelli M. (2019). Piezosurgery in pediatric neurosurgery. World Neurosurg..

[19] Crosetti E., Battiston B., Succo G. (2009). Piezosurgery in head and neck oncological and reconstructive surgery: Personal experience on 127 cases. Acta Otorhinolaryngol. Ital..

[20] Cicciù M., Stacchi C., Fiorillo L., Cervino G., Troiano G., Vercellotti T., Herford A.S., Galindo-Moreno P., Di Lenarda R. (2021). Piezoelectric bone surgery for impacted lower third molar extraction compared with conventional rotary instruments: A systematic review, meta-analysis, and trial sequential analysis. Int. J. Oral Maxillofac. Surg..

[21] Magesty R.A., Galvão E.L., de Castro Martins C., Dos Santos C.R.R., Falci S.G.M. (2017). Rotary instrument or piezoelectric for the removal of third molars: A meta-analysis. J. Maxillofac. Oral Surg..

[22] Ribeiro F.V., Hirata D.Y., Reis A.F., Santos V.R., Miranda T.S., Faveri M., Duarte P.M. (2014). Open-flap versus flapless esthetic crown lengthening: 12-month clinical outcomes of a randomized controlled clinical trial. J. Periodontol..

[23] Desai A., Patil S., Mitra D., Shah R. (2020). Magnetic Mallet-Feel the Future. JIDA J. Indian Dent. Assoc..

[24] Crespi R., Bruschi G.B., Gastaldi G., Capparé P., Gherlone E.F. (2015). Immediate Loaded Implants in Split-Crest Procedure. Clin. Implant. Dent. Relat. Res..

[25] Crespi R., Capparé P., Crespi G., Gastaldi G., Gherlone E.F. (2017). Dimensional Changes of Fresh Sockets with Reactive Soft Tissue Preservation: A Cone Beam CT Study. Implant. Dent..

[26] Menchini-Fabris G.B., Toti P., Crespi G., Covani U., Crespi R. (2020). Distal Displacement of Maxillary Sinus Anterior Wall versus Conventional Sinus Lift with Lateral Access: A 3-Year Retrospective Computerized Tomography Study. Int. J. Environ. Res. Public Health.

[27] Crespi R., Bruschi G.B., Capparé P., Gherlone E. (2014). The utility of the electric mallet. J. Craniofac. Surg..

[28] Crespi R., Capparé P., Crespi G., Gastaldi G., Gherlone E. (2016). Bone-Level Changes around Delayed Dental Implants in Previous Large Bone Defects Filled with Reactive Soft Tissue After Extraction: A Cone Beam Computed Tomography Study. Int. J. Oral Maxillofac. Implant..

[29] Bakkar M., Liu Y., Fang D., Stegen C., Su X., Ramamoorthi M., Lin L.-C., Kawasaki T., Makhoul N., Pham H. (2017). A Simplified and Systematic Method to Isolate, Culture, and Characterize Multiple Types of Human Dental Stem Cells from a Single Tooth. Methods Mol. Biol..

[30] Di Vito A., Giudice A., Chiarella E., Malara N., Bennardo F., Fortunato L. (2019). In Vitro Long-Term Expansion and High Osteogenic Potential of Periodontal Ligament Stem Cells: More Than a Mirage. Cell Transpl..

[31] Schierano G., Baldi D., Peirone B., Mauthe von Degerfeld M., Navone R., Bragoni A., Colombo J., Autelli R., Muzio G. (2021). Biomolecular, Histological, Clinical, and Radiological Analyses of Dental Implant Bone Sites Prepared Using Magnetic Mallet Technology: A Pilot Study in Animals. Materials.

[32] Feher B., Frommlet F., Gruber R., Hirtler L., Ulm C., Kuchler U. (2021). Resonance frequency analysis of implants placed in condensed bone. Clin. Oral Implant. Res..

[33] Giudice A., Bennardo F., Antonelli A., Barone S., Fortunato L. (2020). COVID-19 is a New Challenge for Dental Practitioners: Advice on Patients’ Management from Prevention of Cross Infections to Telemedicine. Open Dent. J..

[34] Giudice A., Antonelli A., Bennardo F. (2020). To test or not to test? An opportunity to restart dentistry sustainably in the ‘COVID-19 era’. Int. Endod. J..

[35] Bennardo F., Antonelli A., Barone S., Figliuzzi M.M., Fortunato L., Giudice A. (2020). Change of Outpatient Oral Surgery during the COVID-19 Pandemic: Experience of an Italian Center. Int. J. Dent..

[36] Chien A.T., Stehle N.E., Karian B.K. (2021). The Use of Chisels in the Extraction of Mandibular Third Molars: A Technique That May Prevent the Aerosolization of Severe Acute Respiratory Syndrome Coronavirus 2. J. Oral Maxillofac. Surg..

[37] Franchignoni F., Salaffi F., Tesio L. (2012). How should we use the visual analogue scale (VAS) in rehabilitation outcomes? I: How much of what? The seductive VAS numbers are not true measures. J. Rehabil. Med..

[38] Devlin H., Horner K., Ledgerton D. (1998). A comparison of maxillary and mandibular bone mineral densities. J. Prosthet. Dent..

